# Comparative Characterization of G Protein α Subunits in *Aspergillus fumigatus*

**DOI:** 10.3390/pathogens9040272

**Published:** 2020-04-09

**Authors:** Yong-Ho Choi, Na-Young Lee, Sung-Su Kim, Hee-Soo Park, Kwang-Soo Shin

**Affiliations:** 1 Department of Microbiology, Graduate School, Daejeon University, Daejeon 34520, Korea; youngho1107@gmail.com (Y.-H.C.); 1209leeny@gmail.com (N.-Y.L.); 2 Department of Biomedical Laboratory Science, Daejeon University, Daejeon 34520, Korea; sungsu@dju.kr; 3 School of Food Science and Biotechnology, Institute of Agricultural Science and Technology, Kyungpook National University, Daegu 41566, Korea; phsoo97@knu.ac.kr

**Keywords:** *Aspergillus fumigatus*, G protein α subunits, asexual development, stress response, antifungal drug, GT, PKA, PKC

## Abstract

Trimeric G proteins play a central role in the G protein signaling in filamentous fungi and Gα subunits are the major component of trimeric G proteins. In this study, we characterize three Gα subunits in the human pathogen *Aspergillus fumigatus*. While the deletion of *gpaB* and *ganA* led to reduced colony growth, the growth of the Δ*gpaA* strain was increased in minimal media. The germination rate, conidiation, and mRNA expression of key asexual development regulators were significantly decreased by the loss of *gpaB*. In contrast, the deletion of *gpaA* resulted in increased conidiation and mRNA expression levels of key asexual regulators. The deletion of *gpaB* caused a reduction in conidial tolerance against H_2_O_2_, but not in paraquat (PQ). Moreover, the Δ*gpaB* mutant showed enhanced susceptibility against membrane targeting azole antifungal drugs and reduced production of gliotoxin (GT). The protein kinase A (PKA) activity of the Δ*ganA* strain was severely decreased and protein kinase C (PKC) activity was detected all strains at similar levels, indicating that all G protein α subunits of *A*. *fumigatus* may be a component of the cAMP/PKA signaling pathway and appear to possess the PKC signaling pathway as an alternative backup pathway to compensate for PKA depletion. Collectively, the three Gα subunits regulate growth, germination, asexual development, resistance to oxidative stress, and GT production differently *via* the PKA or PKC signaling pathway. The function of GanA of *A*. *fumigatus* was elucidated for the first time.

## 1. Introduction

G protein signaling is a universal means of signal transduction in living organisms, activating many G protein coupled receptor (GPCR) mediated cellular processes. The G protein signaling pathway consists of a series of components. External signals are transmitted to target genes *via* GPCR, heterotrimeric G proteins, and various downstream regulators and control numerous regulators of the G protein (RGSs) [1,2,3]. One of the uppermost components of these pathways is the G protein α subunit, which is activated by means of GDP/GTP exchanges and interacts with downstream effectors [1,2,3]. In the model filamentous fungus *Aspergillus nidulans*, three Gα proteins, namely, FadA, GanA, and GanB, were identified [3]. FadA-mediated signaling pathways promote vegetative growth and inhibit conidiation and sterigmatocystin production *via* cAMP-dependent protein kinase A (PKA) [4,5,6]. The FadA-PKA signaling pathway is conserved in most *Aspergillus*, which control toxin synthesis and conidiation [4,7]. GanB forms a functional heterotrimer with SfaD (Gß) and GpgA (Gɤ) and is activated by the GDP/GTP exchange factor RicA [8]. GanB also positively regulates the germination of conidia and negatively controls asexual sporulation [9,10]. The activity of GanB-mediated signaling is negatively regulated by RgsA [11]. The GanB homologue of *A*. *flavus* GpaB function in conidiation, stress responses, toxin production, and virulence [12]. The function of GanA in *A*. *nidulans* is not yet characterized. 

Like *A*. *nidulans*, three predicted Gα proteins, GpaA (Afu1g13140), GpaB (Afu1g12930), and GanA (Afu3g12400) have been identified in the *A*. *fumigatus* genome [13]. GpaA of *A*. *fumigatus* functions as the cognate Gα for FlbA (a regulator of G protein signaling, RGS) and activates vegetative growth while inhibiting asexual development. The dominant activating GpaA^Q204L^ strain causes reduced conidiation and the dominant interfering GpaA^G203R^ mutant restores conidiation [14]. GpaB is a member of the adenylate cyclase stimulating Gα proteins and regulates asexual sporulation *via* activation of cAMP synthesis. GpaB-PkaC1 signaling is involved in the activation of the PKA catalytic subunit PkaC1 and has been proposed to induce both hyphal growth and conidiation [15]. Unlike GpaA and GpaB, the role of GanA in *A*. *fumigatus* is not yet established.

In the present study, to elucidate the function of Gα subunits in *A*. *fumigatus* further, we generated corresponding deletion mutants and investigated the roles of Gα proteins on the growth, development, stress response, and toxin production *via* comparative analyses. 

## 2. Results

### 2.1. Bioinformatic Summary of Gα Subunits

**Figure 1:**
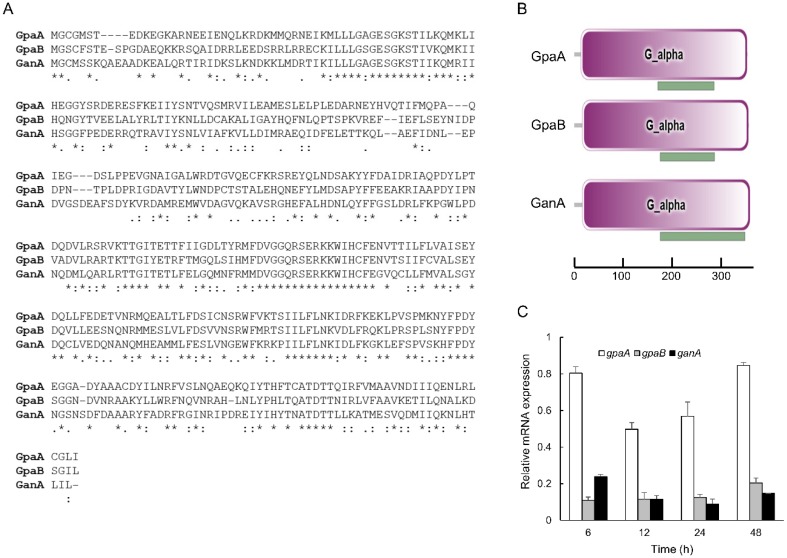
Summary of Gα proteins in *A*. *fumigatus*. (**A**) Multiple sequence alignment of GpaA, GpaB, and GanA proteins using MUSCLE (https://www.ebi.ac.uk/Tools/msa/muscle/). (**B**) Predicted *A*. *fumigatus* Gα proteins are presented schematically using SMART (http://smart.embl-heidelberg.de). Green; ADP ribosylation factor (Arf) domain. (**C**) The mRNA levels of *gpaA*, *gpaB*, and *ganA* throughout the asexual development of wild type (WT).

The ORFs of *gpaA* (Afu1g13140), *gpaB* (Afu1g12930), and *ganA* (Afu3g12400) encodes the protein lengths of 353, 356, and 359 amino acids, respectively in *A. fumigatus* AF293. Based on the protein sequences, three Gα subunits were aligned and compared (Figure 1A). GpaA shares 49.3% and 46.3% identity with GpaB and GanA, and GpaB shares 44.3% identity with GanA. The domain structures of the Gα subunits are very simple and contain only one Gα domain (340, 340, and 343 aa, E-value; 1.24e-221, 3.03e-193, and 2.98e-160) and one GTP-binding ADP ribosylation factor (Arf) domain inside of the Gα domain (170 to 285 aa, 175 to 286 aa, and 176 to 349 aa) (Figure 1B). To characterize the Gα subunits encoding genes, the mRNA levels of *gpaA*, *gpaB*, and *ganA* at different time points in the asexual development were examined, and found that *gpaA* and *ganA* mRNA was highly expressed both early (6 h) and later (48 h) developmental phase. The mRNA levels of *gpaB* was increased at the later developmental phase (Figure 1C).

### 2.2. Generation of Gα Subunit Null Mutants

**Figure 2:**
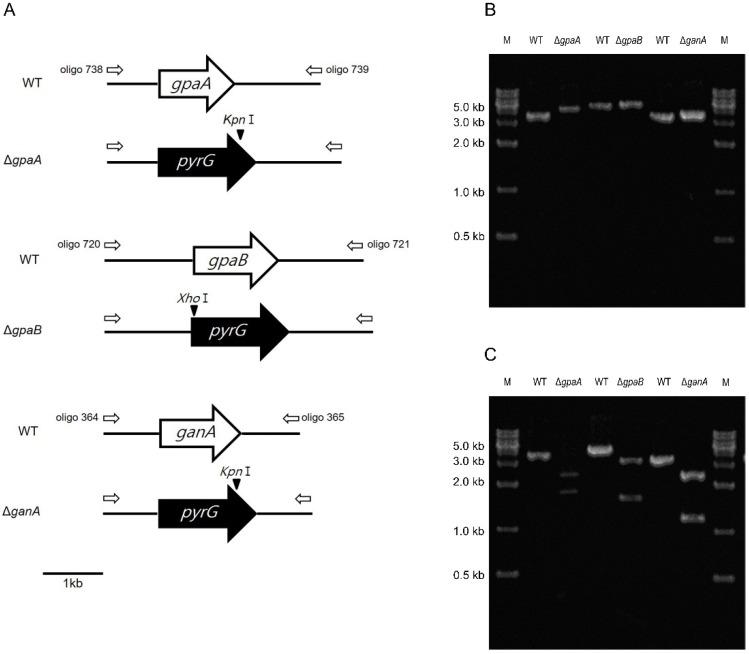
Verification of Δ*gpaA*, Δ*gpaB*, and Δ*ganA*. (**A**) Schematic illustration of the *gpaA*, *gpaB*, and *ganA* regions and restriction maps of WT and mutant strains. (**B**) PCR amplicons for the three strains compared to that of WT. Lane M, molecular weight marker. (**C**) The *Kpn*I (for Δ*gpaA* and Δ*ganA*) and *Xho*I (for Δ*gpaB*) digestion patterns of individual amplicons. While the mutant amplicons are cut into two fragments, the WT amplicon remains uncut.

A targeted gene replacement strategy was used to disrupt the Gα subunit encoding genes from the *A*. *fumigatus* AF293.1 (*pyrG1*) strain [16], with double-joint PCR (DJ-PCR) utilized [17] (Figure 2A). The deletion construct containing the *A*. *nidulans* selective marker (*AnipyrG*^+^) with the 5′ and 3′ franking regions of the Gα subunit encoding genes was introduced into the recipient strain AF293.1 [16]. *AnipyrG*^+^ was amplified from FGSC4 genomic DNA with the primer pair oligo 697/oligo 698. The transformants were isolated and confirmed by means of PCR (Figure 2B) and further confirmed by restriction enzyme digestion (Figure 2C). The oligonucleotides used in this study are listed in Table S1. We also generated relevant complemented strains and found that the cultural phenotypes were similar to those of wild type (WT) (Figure S1).

### 2.3. Gα Subunits Are Involved in Vegetative Growth and Germination

**Figure 3:**
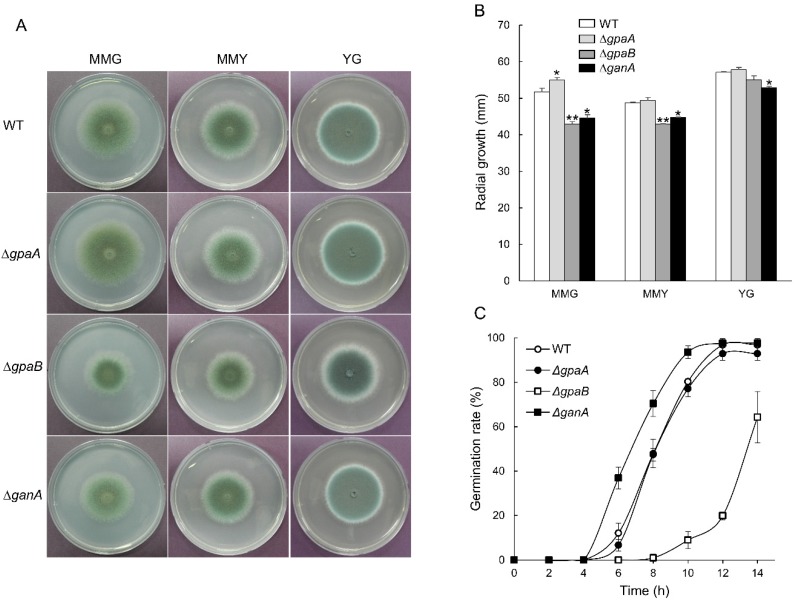
The mutants showed altered vegetative growth and germination. (**A**) Colony photographs of WT, Δ*gpaA*, Δ*gpaB*, and Δ*ganA* strains inoculated on solid MMG (for five days), MMY, and Yeast extract-Glucose (YG) media grown for three days. (**B**) Radial growth rates of WT and three mutant strains. One-way ANOVA with Tukey’s multiple comparison test: * *p* < 0.05, ** *p* < 0.01. (**C**) Kinetics of germ tube outgrowth in WT and mutant strains when inoculated in MMY at 37 °C.

To observe the growth phenotypes of the mutant strains, we point inoculated conidia (1 × 10^5^) of the WT and mutant strains on to MMG, MMY, and YG media, and incubated for three (MMY and YG media) or five days (for the MMG medium) at 37 °C. As shown in Figure 3A, the colony colors were generally differed depending on the mutant strain and culture medium used. Furthermore, while the radial growth rate of the Δ*gpaA* strain was increased in MMG medium, the growth rates of the Δ*gpaB* and Δ*ganA* strains decreased significantly compared to that of WT in the MMG and MMY media (Figure 3B). To investigate the roles of the Gα subunits in controlling spore germination, we analyzed the kinetics of germination in the mutant strains in comparison to that of the WT strain. Excluding the Δ*gpaB* strain, the conidia of all strains began to germinate after 4 h of incubation, and nearly all conidia were germinated at 14 h. However, at 12 h, while approximately 95% of the Δ*gpaA*, Δ*ganA*, and WT strains of conidia germinated, only 20% of the Δ*gpaB* mutant conidia germinated (Figure 3C), suggesting that GpaB positively regulates conidia germination and that the low germination rate affects the decreased mycelial growth. Germination rates of the Δ*ganA* mutant conidia were higher than those of WT conidia at 6, 8, and 10 h of incubation, suggesting that GanA is required for proper conidial germination.

### 2.4. Gα Proteins Regulate Asexual Sporulation

**Figure 4:**
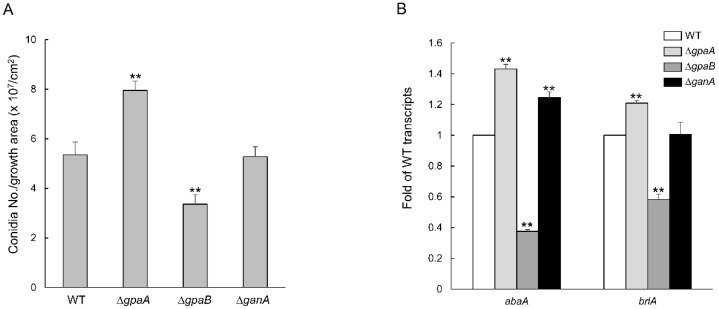
Regulation of asexual development by Gα proteins. (**A**) Conidia numbers produced by WT, Δ*gpaA*, Δ*gpaB*, and Δ*ganA* strains relative to the corresponding growth area (cm^2^). (**B**) Transcript levels of the asexual developmental regulator genes in mutant strains compared to WT as determined by quantitative real time PCR (qRT-PCR). The mRNA levels were normalized using the *ef1α* gene, according to the ΔΔCt method. Data are expressed as the mean ± standard deviation from three independent experiments. One-way ANOVA test: ** *p* < 0.01.

After vegetative growth, *A*. *fumigatus* forms numerous asexual developmental structures including aerial hyphae, conidiophore, metulae, and phialides, which produce chains of conidia. To investigate the roles of Gα proteins in asexual sporulation (conidiation), we carried out quantitative analyses of conidia relative to the growth area on the MMG medium. Conidia production in the Δ*gpaA* mutant (7.95 × 10^7^ conidia/cm^2^) was dramatically increased to a level that was nearly 1.5-fold that of the WT strain, whereas in the case of the Δ*gpaB* strain, the conidia number was significantly lower than in the WT strain (about 0.6-fold) (Figure 4A). We then analyzed the mRNA expression levels of key asexual developmental regulators (*abaA* and *brlA*) compared these results with those of the WT strain, finding that the losses of *gpaA* and *gpaB* showed opposite results. While the loss of *gpaA* resulted in significantly increased levels of the corresponding mRNA, the Δ*gpaB* strain exhibited significantly reduced levels of corresponding mRNA (Figure 4B). These results suggest that GpaA regulates the conidiation and expression of developmental regulators negatively and GpaB is necessary for both proper asexual development.

### 2.5. GpaB Regulates the Oxidative Stress Response

**Figure 5:**
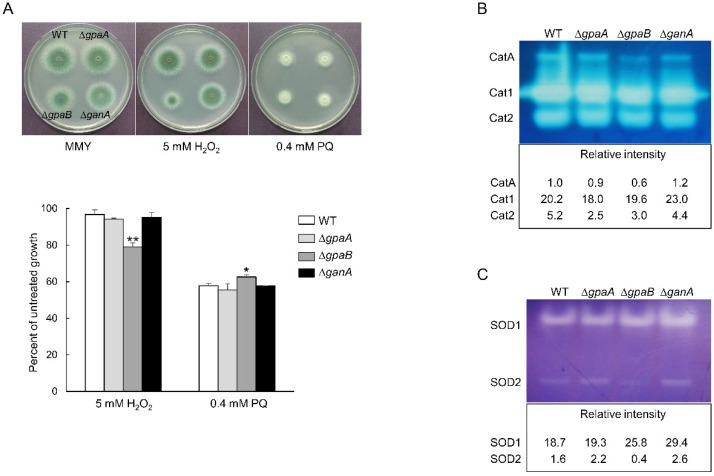
Oxidative stress responses as mediated by Gα proteins. (**A**) Radial growth of the WT, Δ*gpaA*, Δ*gpaB*, and Δ*ganA* strains in the presence of the oxidative stressors H_2_O_2_ (5 mM) and paraquat (PQ, 0.4 mM) following incubation at 37 °C for 48 h. (**B**) Catalase and (**C**) SOD activities of WT, Δ*gpaA*, Δ*gpaB*, and Δ*ganA* strains shown in non-denaturing polyacrylamide gels.

To test whether Gα proteins are associated with stress responses, the growth of the mutant strains was determined under a variety of stressors. The mutants exhibited no altered tolerance to the cell wall stressors Calcofluor white and Congo red (data not shown). In the treatment of oxidative stressors, the Δ*gpaB* mutant was significantly sensitive to hydrogen peroxide (H_2_O_2_), growth was inhibited approximately 20% compared to an untreated control. However, the Δ*gpaB* mutant showed more tolerance against paraquat (PQ) (Figure 5A). To determine the potential contribution of GpaB to oxidative stress, we analyzed the activity of catalases and superoxide dismutases (SODs). Among three catalases, the activity of the conidia specific catalase CatA and mycelial catalase Cat2 were reduced by the loss of *gpaB* (Figure 5B). While the activity of cytoplasmic Cu/Zn SOD (SOD1) was increased in the Δ*gpaB* and Δ*ganA* strain, the mitochondrial Mn SOD (SOD2) activity was reduced in the Δ*gpaB* strain (Figure 5C). These data indicate that GpaB plays a protective role against oxidative stress and it is exerted through CatA, Cat2, and SODs.

### 2.6. Gα Subunits Positively Govern Resistance to Membrane Targeting Antifungal Drugs

**Figure 6:**
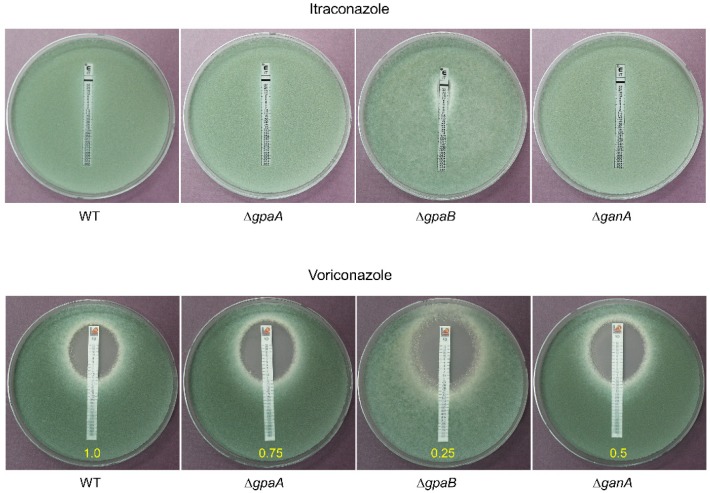
Deletion of Gα proteins reduce resistance against azole antifungal drugs. The effects of azole antifungal agents on the growth of WT and mutant strains were investigated with E-test strips. MIC values were presented as yellow.

To investigate the involvement of Gα subunits in governing the response to membrane targeting antifungal drugs, we tested the susceptibility of WT and mutant strains to the azole class of antifungal drugs using E-Test strips. As shown in Figure 6, Itraconazole failed to inhibit all tested strains except the Δ*gpaB* strain. The mutant strains were more susceptible to Voriconazole than the WT strain. While the Voriconazole MIC was 1.0 µg/mL for the WT strain, the MICs for the Δ*gpaA*, Δ*gpaB*, and Δ*ganA* strains were 0.75, 0.25, and 0.5 µg/mL, respectively. The Δ*gpaB* strain was the most susceptible to azole antifungal drugs. These results suggesting that Gα proteins positively regulate resistance against membrane targeting azole drugs.

### 2.7. Gα Subunits Are Involved in Gliotoxin Production and Protection

**Figure 7:**
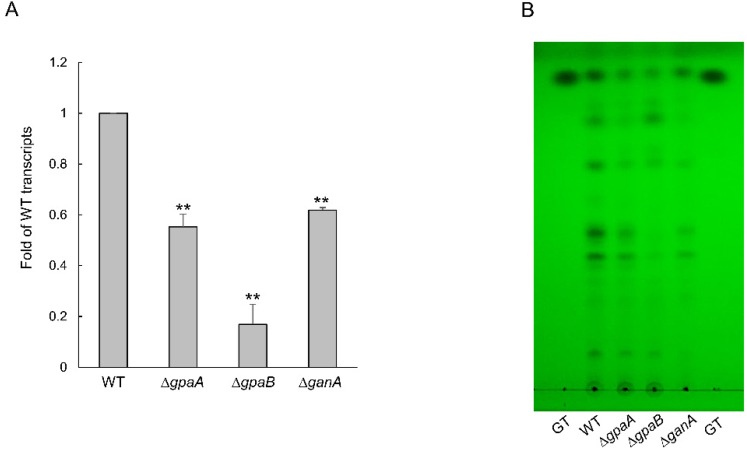
The roles of Gα proteins in gliotoxin (GT) production and protection. (**A**) qRT-PCR analysis of *gliZ* gene in mutant strains compared to WT. (**B**) Determination of GT production in WT and mutant strains. The culture supernatant of each strain was extracted with chloroform and subjected to TLC. Statistical differences were evaluated with ANOVA tests. ** *p* < 0.01.

Gliotoxin (GT) is the most important secondary metabolite and is regulated by G protein signaling. As Gα proteins are the main components in relation to G protein signaling, we examined the GT production of Gα protein encoding gene deletion mutants. The expression level of GT biosynthetic transcription factor *gliZ* mRNA was significantly lower in the Δ*gpaB* strain (0.17 to 0.33-fold) than in other mutant and WT strains (Figure 7A). Accordingly, we assessed the levels of GT itself in the indicated strains, finding that all mutants produced smaller amounts of GT (Figure 7B).

### 2.8. Gα Subunits Control Protein Kinase A (PKA) and Protein Kinase C (PKC) Activity

**Figure 8:**
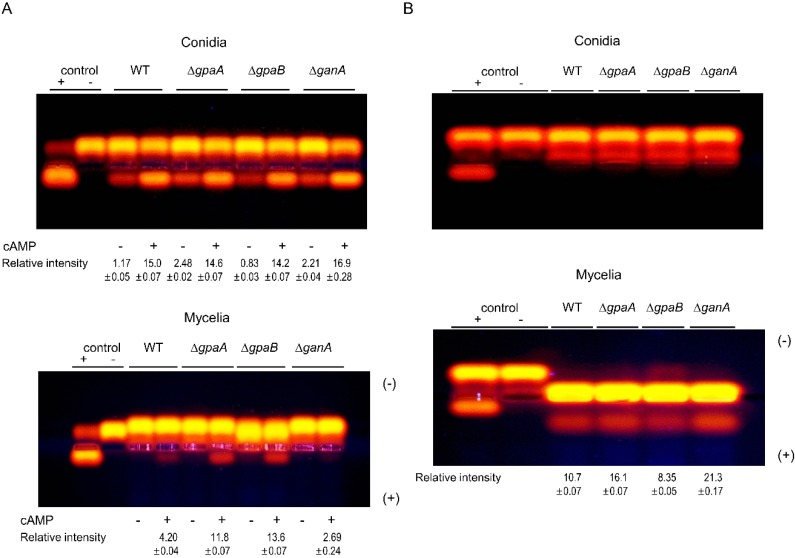
Gα proteins regulate protein kinase A (PKA) and protein kinase C (PKC) activity. (**A**) PKA activity levels of four strains as monitored by gel electrophoresis. (**B**) PKC activity of WT and mutant strains. A phosphorylated substrate migrates toward the anode (+). Conidial and mycelial extracts were analyzed.

In the model fungus *A. nidulans*, G protein signaling pathways are involved in cAMP/PKA signaling pathway [3]. G proteins α subunits are known to regulate cAMP/PKA signaling pathway by regulated the adenylate cyclase activity. To determine whether Gα subunits are involved in cAMP/PKA signaling in *A*. *fumigatus*, the PKA activity was assessed using fluorescent kemptide (Promega, Madison, WI, USA) as a PKA substrate. In conidial extracts, all strains exhibited PKA activity regardless of presence of cAMP and Δ*ganA* strain showed highest activity. The Δ*gpaA* and Δ*gpaB* strains exhibited very high PKA activity levels compared to that of the WT strain in the presence of cAMP. In contrast, the Δ*ganA* strain showed almost negligible detectable PKA activity in mycelial preparations (Figure 8A). We also attempted to detect the effects of Gα subunits on PKC activity with PepTag Non-Radioactive PKC Assay Kit (Promega, USA). PKC activities were not detected in conidial preparation because PKC was localized in septa and cell wall of growing hyphae [18]. All strains tested presented PKC activity in mycelial extracts, and the highest level of activity was found in the Δ*ganA* strain (Figure 8B). These results indicate that the G protein α subunit GanA likely plays a role in regulating PKA activity. 

## 3. Discussion

The G protein signaling of *Aspergillus* functions during vegetative growth, asexual development, the stress response, and virulence in humans [1,2,3,19,20]. G protein α subunits play important roles as an upstream signal intermediator and regulate asexual sporulation and germination [9,13,15,21]. To elucidate more profound roles of Gα subunits, in this study we comparatively characterized the Gα subunits GpaA, GpaB, and GanA in *A*. *fumigatus*.

Previously, Mah and Yu generated the dominant activating (GpaA^Q204L^) or interfering (GpaA^G203R^) mutants and found that GpaA of *A*. *fumigatus* positively regulates vegetative growth but inhibits conidiation and functions as a primary target of FlbA [14]. Similar to the phenotypes of the dominant interfering (GpaA^G203R^) mutant strain, the Δ*gpaA* mutant strain exhibited increased conidia production (Figure 4A). In addition, we newly found that deletion of *gpaA* increased mRNA levels of central regulatory genes for conidiation including *abaA* and *brlA*, implying that the GpaA singling can repress asexual development *via* negative regulation of *brlA*. In *A. nidulans*, the FadA (GpaA homologue) repress conidia *via* PKA activity [22]. These results demonstrated that the role of GpaA signaling in conidiation is conserved in two *Aspergilli*.

Research has also suggested that GpaB appears to be involved in vegetative growth and conidiation *via* PkaC1 signaling [15]. Our study also found that the Δ*gpaB* strain similarly showed reduced vegetative growth and conidiation. Interestingly, the germination rate of the Δ*gpaB* mutant was significantly lower than those of other strains (Figure 3C), suggesting that the reduced radial growth may be due to the lower germination rate. Unlike our results, the phenotype of the Δ*gpaB* mutant in *A. flavus* is slightly different. The colony size of the Δ*gpaB* mutant was increased compared to that of WT and complemented strains [12], suggesting that the roles of GpaB may not be conserved in *Aspergillus*.

With regard to the fungi response against oxidative stresses by the enhanced expression of the SOD and catalase, the SOD activity was increased by the treatment of PQ and the catalase activity was enhanced by exogenous H_2_O_2_ [23]. Therefore, tolerance against oxidative stress was detected with the growth rate and activity of the SOD and catalase. The tolerance to oxidative stress was similar to those of all strains tested here except the Δ*gpaB* mutant. The Δ*gpaB* mutant displayed less growth under oxidative stress generated by 5 mM H_2_O_2_ and showed low activity of conidia specific catalase CatA and mycelial catalase Cat2, which may confer conidial tolerance against H_2_O_2_. Tolerance against another oxidative stressor, PQ, showed an opposite trend. The growth was increased at 0.4 mM PQ and the activity of cytoplasmic SOD1 was high in this condition due to the loss of *gpaB* (Figure 5). However, the activity of SOD2 (mitochondrial Mn SOD) was reduced. It has been reported that SOD2 plays a major role in the protection against high temperatures [24]. These data indicate that GpaB is involved in oxidative stress responses in this fungus.

In addition, we tested the growth and MIC of WT and mutant strains in the presence of azole antifungal agents and found that mutant strains were highly susceptible to azole class agents (Figure 6), indicating G protein α subunits play key roles in governing the resistance to azole drugs and a better understanding of downstream signaling pathways would open up new research avenues to the development of enhanced therapeutic measures for fungal infections.

In some *Aspergillus* species, the G protein signaling pathway plays an important role in secondary metabolism [3]. In *A*. *nidulans*, the G protein mediates cAMP signaling are required for sterigmatocystin biosynthesis [11]. In *A*. *flavus*, The Gα subunit GpaB is also essential for the biosynthesis of aflatoxins, and aflatoxin production was drastically inhibited by the loss *gpaB* [12]. Despite the small difference in the amounts, the Gα encoding gene deletion mutants of *A*. *fumigatus* produced less GT than the WT strain. The collection of these results proposes that Gα subunits are important components in the production of secondary metabolites.

The GpaA homologue of *A*. *nidulans* FadA is involved in the activation of cAMP dependent protein kinase A (cAMP/PKA) [10,25]. Deletion of the PKA catalytic subunit *pkaA* led to hyperactive conidiation with restricted vegetative growth, and the overexpression of *pkaA* caused reduced conidiation and enhanced vegetative growth [25]. GpaA of *A*. *fumigatus* paired with RGS FlbA and had a role in vegetative growth and asexual development. The dominant interfering *gpaA* mutant caused reduced hyphal growth and increased conidiation [14]. GpaB also participates in the cAMP/PKA signaling pathway. Deletion of *gpaB* results in reduced conidiation and decreased activation of adenylate cyclase [15]. To verify these findings, we detected the PKA and PKC activities of mutant and WT strains. Mycelial extracts of the Δ*gpaA* and Δ*gpaB* strains exhibited significant PKA activity only in the presence of cAMP (Figure 8A). This result suggests that PKA exists but is not activated in these mutant strains, likely due to the lack of significant amounts of cAMP and confirming that GpaA and GpaB are components of the cAMP/PKA signaling pathway. However, PKA activity was scarcely detected in the G protein α subunit mutant strain, Δ*ganA* irrespective of cAMP (Figure 8A), indicating that PKA may be severely decreased by the absence of *ganA*. PKC activity was detected in the protein extracts of all mutant strains, and the highest level was detected in the Δ*ganA* strain (Figure 8B). From this finding, we proposed that all G protein α subunits of *A*. *fumigatus* may be a component of the cAMP/PKA signaling pathway and may possess the PKC signaling pathway as alternative backup pathway to compensate for PKA depletion. Understanding of GanA-mediated signaling and the PKC signaling that are associated with the development of *A*. *fumigatus* will require further investigation.

In conclusion, we characterized the role of genes encoding for Gα subunits in the human pathogen *A*. *fumigatus*. Our results demonstrated that the roles GpaA, GpaB, and GanA are slightly different, but play crucial roles in fungal growth, development, and secondary metabolism through the PKA or PKC signaling pathway. Additional experiments for dissecting the downstream effects of the G protein signaling pathway will be needed to understand how the Gα proteins play different roles in *A. fumigatus*.

## 4. Materials and Methods

### 4.1. Strains and Culture Conditions

Glucose minimal medium (MMG) and MMG with 0.1% yeast extract (MMY) were used for the culturing of wild type (WT, AF293) and Gα encoding gene null mutant strains [26,27]. The strains used in this study are listed in Table 1. To examine the production of secondary metabolites, conidia of relevant strains were inoculated at a final concentration of 5 × 10^5^ conidia/mL to 50 mL of liquid MMY and incubated at 250 rpm at 37 °C for four days.

### 4.2. Nucleic Acid Manipulation and Quantitative RT-PCR

Total RNA isolation and quantitative RT-PCR (qRT-PCR) assays were conducted as previously described [14]. Briefly, conidia (5 × 10^5^ conidia/mL) of the WT and mutant strains were inoculated into MMY medium and incubated at 37 °C for 3 days or 4 days (for *gliZ*). Individual colonies were then collected and squeeze-dried. Each sample was homogenized using a Mini Bead beater in the presence of the TRIzol^®^ reagent (Invitrogen, Carlsbad, CA, USA) and silica/zirconium beads (BioSpec Products, Bartlesville, OK, USA). QRT-PCR assays were performed according to the manufacturer’s instructions using a Rotor-Gene Q device (Qiagen, Germantown, MD, USA). For the qRT-PCR process, the One Step RT-PCR SYBR Mix (MGmed, Seoul, Korea) was used. The primers used in this experiment are listed in Table S1. The PCR conditions were 95℃ for 5 min followed by 95 and 55℃/30 s for 40 cycles. Amplification of one single specific target DNA was checked by a melting curve analysis. The expression ratios were normalized to the *ef1α* expression level and calculated according to the ΔΔCt method [29].

### 4.3. Phenotype Analyses

To examine the germination levels, conidia of the WT and mutant strains were inoculated in liquid MMY and incubated at 37 °C, and germ tube formation outcomes were examined every 2 h after inoculation under a microscope. For an oxidative stress test, hydrogen peroxide (5 mM) and paraquat (0.4 mM) were added to the MMY media. The effects of azole antifungal agents to the growth of WT and mutant strains were investigated with E-test strips (Biomérieux, Durham, NC, USA) impregnated with a gradient of Iitraconazole or Voriconazole. Conidial suspensions (1 × 10^4^ conidia) were inoculated to solid medium and E-test strips placed on the plate. After incubation at 37 °C for 48 h, minimal inhibitory concentrations (MIC) values were determined as the zone edge intersect the strips. The production of gliotoxin (GT) was determined, as described previously [30], and the GT standard was purchased from Sigma-Aldrich (USA).

### 4.4. Measurements of Enzyme Activity

A freeze-dried sample was used for total protein extraction following a protocol previously described [31]. Catalase activity on gels was detected by the potassium ferricyanide reagent [32] and SOD activity was visualized by negative staining using nitro blue tetrazolium (NBT, Sigma-Aldrich, St. Louis, MO, USA) according to the method of Beauchamp and Fridovich [33]. To measure the PKA and PKC activities, PepTag^®^ Non-Radioactive cAMP-Dependent Protein Kinase Assay kit and PepTag^®^ Non-Radioactive PKC Assay Kit (Promega, USA) were used.

### 4.5. Statistical Analyses

Comparisons of mRNA expressions, radial growth, and viability within the different strains were performed by one-way ANOVA and adjusted with Tukey’s multiple comparison tests. All results are expressed as the mean ± standard deviation (SD), and a *p* value of less than 0.05 was considered statistically significant. The experiments were performed with three replicates for the indicated strain and were repeated three times.

## Figures and Tables

**Table 1:** *A*. *fumigatus* strains used in this study.

Strain	Genotype	Reference
*A*. *nidulans* FGSC4	*veA*^+^ (Wild type)	FGSC ^a^
AF293	Wild type	[28]
AF293.1	*pyrG1*	[16]
Δ*gpaA*	*pyrG1* Δ*gpaA*::*AnipyrG*^+^	This study
Δ*gpaB*	*pyrG1* Δ*gpaB*::*AnipyrG*^+^	This study
Δ*ganA*	*pyrG1* Δ*ganA*::*AnipyrG*^+^	This study

^a^ FGSC, Fungal Genetics Stock Center.

## Supplementary Materials

The following are available online at https://www.mdpi.com/2076-0817/9/4/272/s1, Figure S1: Colony morphologies of WT, mutant, and complemented strains, Table S1: The oligonucleotides used to in this study.
