# Supplementary material for: Comparative Characterization of G Protein α Subunits in Aspergillus fumigatus
Source: Pathogens. 2020 Apr 9;9(4):272. doi: 10.3390/pathogens9040272 (PMC7238038; doi:10.3390/pathogens9040272)
Supplement: Supplementary file 1 [file pathogens-09-00272-s001.pdf]

pathogens

Article

Comparative Characterization of G Protein α Subunits in *Aspergillus fumigatus*

Yong-Ho Choi [1], Na-Young Lee [1], Sung-Su Kim [2], Hee-Soo Park [3] and Kwang-Soo Shin [1,*]

[1] Department of Microbiology, Graduate School, Daejeon University, Daejeon 34520, Korea; youngho1107@gmail.com (Y.-H.C.), 1209leeny@gmail.com (N.-Y.L.)

[2] Department of Biomedical Laboratory Science, Daejeon University, Daejeon 34520, Korea; sungsu@dju.kr

[3] School of Food Science and Biotechnology, Institute of Agricultural Science and Technology, Kyungpook National University, Daegu 41566, Korea; phsoo97@knu.ac.kr

* Correspondence: shinks@dju.kr

Received: 12 February 2020; Accepted: 03 April 2020; Published: date

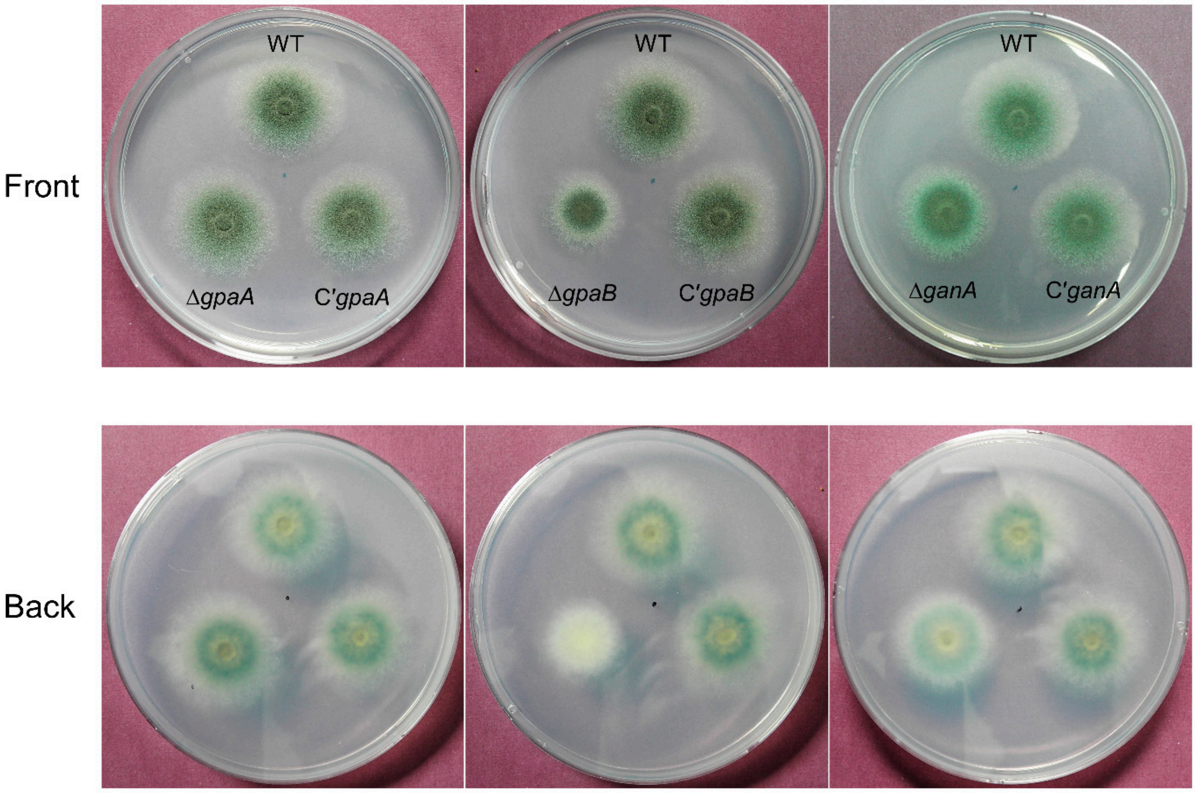

Figure S1. Colony morphologies of WT, mutant, and complemented strains. Each strain was inoculated on solid MMG with supplements and grown for 3 days.

Table S1. Oligonucleotides used in this study.

Name	Sequence (5'→3')	Purpose
oligo697	GCAATGTAAAGCTAACGTGCGTG	5' *AnpyrG* marker
oligo698	TGCCTTTAAGCTTCGGGTAGAG	3' *AnpyrG* marker
oligo710	CCCACCCCCAAATCTACCTA	5' flanking region of *gpaA*
oligo1030	*TTTGTAGGCTTTGGGCTGTTCACAA*CTTGGCGACAGTTGACGATTCAATT	5' *gpaA* with *AnpyrG* tail
oligo1031	*CTGATCTACCCCTTGGAACGCAGCA*TGAGCCATTATACTTGACGGAGTGA	3' *gpaA* with *AnpyrG* tail
oligo713	ATCATCCGCATCAGAAGGGA	3' flanking region of *gpaA*
oligo738	CCACCTCCCACTTTTTCTCC	5' nested of *gpaA*
oligo739	GGCGAGGCTGAATTCGAGAT	3' nested of *gpaA*
oligo716	GTTGGTGGCCGTATCGGGAC	5' flanking region of *gpaB*
oligo1032	*TTTGTAGGCTTTGGGCTGTTCACAA*AACAGCGGAAAGGCCTAAAG	5' *gpaB* with *AnpyrG* tail
oligo1033	*CTGATCTACCCCTTGGAACGCAGCA*TCATTCCAATCGTTCCATCA	3' *gpaB* with *AnpyrG* tail
oligo719	AAGAGCGAGGGGGAGAGTGA	3' flanking region of *gpaB*
oligo720	GCCCAAGTCACCATGGTCTG	5' nested of *gpaB*
oligo721	GGGCCCACCGTTATGCTCTA	3' nested of *gpaB*
oligo360	CTCTGTTTCCCCTCCACAAA	5' flanking region of *ganA*
oligo1034	*TTTGTAGGCTTTGGGCTGTTCACAAGGACAA*AGCGTGGAGATAGG	5' *ganA* with *AnpyrG* tail
oligo1035	*CTGATCTACCCCTTGGAACGCAGCA*ACCTGAGACGCCAAATTTTC	3' *ganA* with *AnpyrG* tail
oligo363	GACAACCCAGCTGATGACTGT	3' flanking region of *ganA*
oligo364	GTGCTCGAACTCCTTGCTCT	5' nested of *ganA*
oligo365	ACGGCTGAGCTTCAAGACAT	3' nested of *ganA*
oligo699	CCATGTGTGTCGAGTCCTTC	5' *ef1α* for qRT-PCR normalization
oligo700	GAACGTACAGCAACAGTCTGG	3' *ef1α* for qRT-PCR normalization
oligo256	TTCCAAGCAGAGCTTGTCAC	5' *brlA* for qRT-PCR
oligo257	CCAGGTTCTTTGCACTTGAA	3' *brlA* for qRT-PCR
oligo303	GCTACCACTCTGCATCCTCA	5' *abaA* for qRT-PCR
oligo304	TACGAGCTCCAGCATGATTC	3' *abaA* for qRT-PCR
oligo271	AAATCCATCACATCCACCCT	5' *gliZ* for qRT-PCR
oligo272	GGTTGTTCATGGTCAGTTGC	3' *gliZ* for qRT-PCR
oligo1462	ACGTACCGCATGTTTGATGT	5' *gpaA* for qRT-PCR
oligo1463	CAGCTGATCGTACTCCGAGA	3' *gpaA* for qRT-PCR
oligo1464	CTCTGGAATGATCCATGCAC	5' *gpaB* for qRT-PCR
oligo1465	CAAAGAAATATGGCGCAGAA	3' *gpaB* for qRT-PCR
oligo1466	CCCGTTTAAGGACAACTGGT	5' *ganA* for qRT-PCR
oligo1467	ACCAACGTCCATCATTCTGA	3' *ganA* for qRT-PCR

© 2020 by the authors. Submitted for possible open access publication under the terms and conditions of the Creative Commons Attribution (CC BY) license (http://creativecommons.org/licenses/by/4.0/).
